# The prevalence of multimorbidity in virally suppressed HIV-positive patients in Limpopo

**DOI:** 10.4102/sajhivmed.v24i1.1495

**Published:** 2023-09-18

**Authors:** Limakatso Lebina, Tumiso Malatji, Firdaus Nabeemeeah, Kegaugetswe Motsomi, Tsundzukani Siwelana, Khuthadzo Hlongwane, Neil Martinson

**Affiliations:** 1 Perinatal HIV Research Unit, SAMRC Soweto Matlosana Collaborating Centre for HIV/AIDS and TB, University of the Witwatersrand, Johannesburg, South Africa; 2 Clinical Research Department, Africa Health Research Institute, Durban, South Africa; 3 Department of Public Health Medicine, University of Limpopo, Polokwane, South Africa; 4 Center for TB Research, Johns Hopkins University, Baltimore, United States of America

**Keywords:** NCD, PLWH, hypertension, diabetes, chronic diseases

## Abstract

**Background:**

Non-communicable diseases (NCDs) are an emerging global public health problem.

**Objectives:**

To assess the prevalence of NCDs and their risk factors among adults on antiretroviral therapy (ART).

**Method:**

This was a cross-sectional study (July 2019 – January 2020) in Limpopo, South Africa. Patients were enrolled if they were ≥ 40 years, HIV-positive, and virologically suppressed on ART. Data were analysed descriptively, and a binomial regression model was used to identify risk factors for NCDs.

**Results:**

The majority of participants (65%; 319/488) were women. Most (83%; 405/488) were aged 40–59 years; 60% (285/472) were overweight or obese. Based on self-report, 22% (107/488) were currently smokers. Almost half (44%) 213/488) reported daily consumption of vegetables and 65% (319/488) exercised regularly and 39% (190/488) reported treatment for another chronic disease. The leading comorbid conditions were hypertension (32%; 158/488) and diabetes mellitus (5%; 24/488). Risk factors for hypertension included age 60 years and older (relative risk [RR]: 1.72; 95% confidence interval [CI]: 1.29–2.30) diabetes (RR: 1.42; 95% CI: 1.08–1.87), overweight (RR: 1.32; 95% CI: 1.03–1.69) and obesity (RR: 1.69; 95% CI: 1.32–2.17).

**Conclusion:**

There is a high prevalence, both of risk factors for NCDs and multimorbidity (> 1 chronic disease) in patients who are ≥ 40 years and virologically suppressed on ART.

**What this study adds:** There are limited data on the prevalence and risk factors for NCDs among adults on ART in low- and middle-income countries. Integrated chronic diseases management needs to be informed by local data.

## Background

HIV and AIDS remain the leading public health problem in many countries due to associated morbidity and mortality.^1^ In 2020, an estimated 7.8 million children and adults were living with HIV and AIDS in South Africa, and the HIV prevalence among adults aged 15–49 years was 19%.^2^ However, approximately 66% of people living with HIV (PLWH) in South Africa are virologically suppressed on antiretroviral medication.^2^

Similarly, there is an increasing burden of non-communicable diseases (NCDs) in low- and middle-income countries (LMIC) in settings with a high burden of HIV and/or AIDS and tuberculosis (TB).^3,4,5^ Some of the explanations are the complex interactions of risk factors, management and health outcomes between communicable diseases (CDs) and NCDs.^6^ For example, tobacco use is a risk factor for TB, and people with diabetes have a higher risk of contracting TB.^7^ In addition, LMIC are confronted with other social determinants of health such as low health literacy and weak healthcare systems.^8^

In South Africa, the population prevalence of hypertension in adults 18 years and older is 27% and that of diabetes is 3%.^9,10^ Consequently, there is a high prevalence of multimorbidity in patients receiving care in primary healthcare clinics, accounting for 45% of all prescription visits in some settings.^11^ A systematic review of multimorbidity (coexistence of two or more chronic diseases in an individual) in South Africa reported that the prevalence of multimorbidity varies depending on the age of the cohort from 3% – 23% in younger to 30% – 87% in older adults.^12^

Moreover, some antiretroviral therapy (ART) increases the risk for chronic comorbidities such as diabetes, metabolic abnormalities, depression and cardiovascular diseases.^13,14,15,16,17,18,19^ The prevalence of chronic diseases comorbidities increases with age: in one cohort in the United States it increased from 8.2% in 2000 to 22.4% in 2009.^20^ Chronic comorbidities are also associated with higher healthcare utilisation, reduced functional capacity, and depression.^21,22^ The high prevalence of other chronic diseases among PLWH receiving antiretroviral medication complicates disease management and quality of life, and impacts survival.^23^

Many patients in South Africa access chronic medication through public health facilities.^24^ One of the strategies recommended in the management of PLWH is to offer integrated HIV treatment and care and other health services that optimise health and well-being of PLWH.^25^ South Africa has developed and implemented guidelines to support the integration of HIV services with other chronic diseases.^26,27^ Many health systems, especially in LMIC, have largely focused on CDs, resulting in unstructured approaches to NCDs in clinical care and adaptations are required to address the growing prevalence of multimorbidity.^28^

There is a need for increased surveillance for other chronic diseases, especially NCDs, among PLWH in LMIC to inform strategies on integrated HIV/CD care models or programmes.^15^ Therefore, we assessed the prevalence of NCDs and their risk factors in a cohort of HIV-positive adults ≥ 40 years, virologically suppressed in the Limpopo province.

## Methods

This was a cross-sectional study that included a health questionnaire and medical records review in patients attending chronic disease clinic in two (primary healthcare and a tertiary hospital) health facilities. The study was conducted in Capricorn district, Limpopo province, South Africa, between July 2019 and January 2020.

### Study setting

The Capricorn district of Limpopo province has an estimated population of 1 350 111 in 2019.^29^ The estimated overall HIV prevalence in Limpopo province was 8.9% in 2017, with 17.2% of people aged 15–49 years.^30^ The proportion of patients remaining on ART in Capricorn district was 73% in 2019/2020, of whom 86% were virologically suppressed.^29^ Overall, in those ≥ 65 years, over 70% of deaths are due to NCDs, while for those aged 35–64 years, approximately 30% of deaths are related to HIV and TB.^29^

In the Capricorn district, there is a total of six state hospitals, two of which are tertiary hospitals, and several primary healthcare facilities. Based on the data from primary healthcare facilities ideal clinic assessments, 76% of clinics in the Capricorn district had achieved ideal clinic status.^29^ Participants for this study were recruited from Rethabile primary healthcare clinic and Mankweng Hospital ARV programmes.

### Study procedures

Trained study team members approached patients attending ARV clinic in the waiting areas to give study information. If a patient was interested in participating in the study, a detailed description of the study, risks and benefits and study activities was provided, followed by individual informed consent. Consented participants were then assessed for eligibility to be enrolled in study, provided they were virologically suppressed, aged 40 years and older and had been receiving care for HIV in that clinic for more than 6 months. Virally suppressed PLWH were selected as they represent the majority of PLWH. Adults, 40 years and older, were selected because as PLWH live longer, this group will most likely be the group that, similar to their peers without HIV, will bear the biggest burden of NCDs.

Enrolled participants completed a health questionnaire administered by the study team. This included demographics, detailed smoking, alcohol, physical activity, and diet history. The participants were asked questions regarding their diet such as frequency they ate fruits and vegetables, usage of salt in meal preparations and consumption of sweetened beverages. In addition, all participants had anthropometric measures (weight, height, waist circumference) done if not already recorded in the medical records. Measurements were carried out by trained study personnel in a standardised manner. Weight was measured using a calibrated digital scale and with the participant in light clothing, without shoes and with pockets emptied. Height was recorded in centimetres to one decimal place using a stadiometer. Using a non-elastic tape, waist circumference was measured in centimetres at the narrowest part of the torso, midway between the lowest rib and the iliac crests.

Trained health professionals conducted the abstraction of data such as date of start of ARV medication, current and previous regimens, results of previous investigations, other medical conditions and medication taken for those conditions from participants’ medical records.

In this study, body mass index (BMI), calculated as weight (kg)/height (m^2^), was categorised as follows: underweight (< 18.5), normal (18.5–24.9), overweight (25.0–29.9) and obese (≥ 30.0).^31^ Medical history of hypertension on records review, and any blood pressure reading above 140/90 mmHg on the date of assessment was considered as high blood pressure (hypertension).^32^ Diabetes mellitus and all other conditions diagnosis was based on medical records review.

The participants’ medical records were then reviewed, and date of start of ART, current and previous regimens, results of previous investigations, other medical conditions and medication taken were abstracted.

### Sample size and data collection

The sample size was calculated based on an estimated prevalence of hypertension among HIV-positive patients as the primary outcome measure. Because of the unavailability of studies in Limpopo, the anticipated primary outcome was estimated at 0.50, with a 5% acceptance margin of error. We planned to enrol 700 participants across the two study clinics.

Data were collected by the study team on an electronic database, REDCap^®^.

### Statistical analysis

Descriptive statistics such as medians and interquartile ranges (IQR) were calculated for continuous variables like age. Frequencies and their proportions for categorical variables were determined and stratified by age groups (40–49 years; 50–59 years and 60 years and older), as well as gender and blood pressure status where appropriate.

Fisher’s exact test or *χ* ^2^ was utilised to assess for statistical significance for categorical variables stratified by age. Risk factors such as age, gender, race, BMI, and a diagnosis of diabetes associated with hypertension were evaluated using univariate regression where the parameter estimate, standard error, 95% confidence interval and *P*-values were determined. Risk factors that were associated with hypertension such as age, level of education, employment status and BMI in the univariate model were included in the multivariate analysis model.

All statistical analysis was conducted in SAS^®^ Enterprise Guide V.7.1 (SAS Institute) using SAS/STAT.

## Results

**FIGURE 1:**
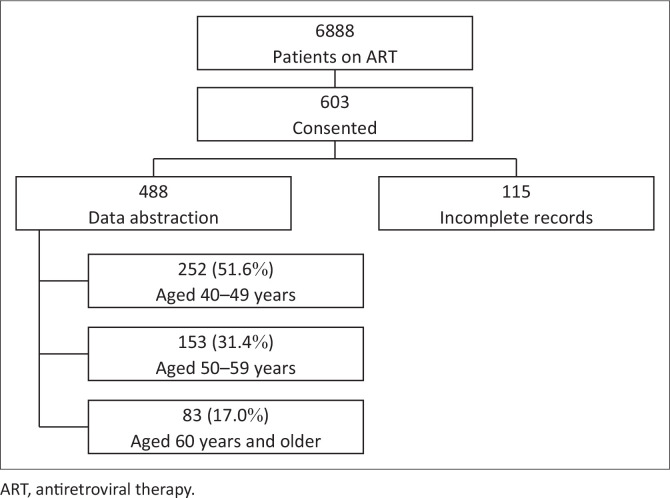
Diagrammatic representation of participants enrolled and analysed on the prevalence of multimorbidity in virally suppressed HIV-positive patients.

A total of 6888 patients were accessing on ART at the two study sites; 603 (8.8%) provided informed written consent to be part of the study, and each completed the health questionnaire. Nineteen percent (*n* = 115) of enrolled participants were not included in the analysis due to substantial missing data on ART (Figure 1). A total of 488 patients are therefore included in this analysis.

### Baseline characteristics

The median age of participants was 49 years (IQR: 45; 57 years). Most (252/488; 51.6%) of the participants were between the ages of 40–49 years, and only 17% (83/488) were 60 years and older (Figure 1). Overall, 65.4% (319/488) were women (Table 1). More participants described their residential area as semi-urban (52.9%), while 47.1% indicated that they lived in a rural area. The level of highest attained education was lower among those aged 60 years and older, with 40% (33/83) who had no school or had not completed primary school and 8% (7/83) who had completed tertiary level education. Overall, 12% (60/488) of participants were retired or pensioners, 32% (158/488) were unemployed and 55% (270/488) were employed.

**TABLE 1:** Demographic characteristics of participants enrolled in the study of the prevalence of multimorbidity in virally suppressed HIV-positive patients.

Variable	Overall (*N* = 488)		40–49 years (*n* = 252)		50–59 years (*n* = 153)		60+ years (*n* = 83)	
	*n*	%	*n*	%	*n*	%	*n*	%
**Gender**								
Male	169	35	76	30	59	39	34	41
Female	319	65	176	70	94	61	49	59
**Area**								
Peri-urban	258	53	154	61	78	51	26	31
Rural	230	47	98	39	75	49	57	69
**Highest level of education and employment status**								
No school or primary school not completed	58	12	9	4	16	10	33	40
Completed primary but not secondary school	221	45	104	41	81	53	36	43
Secondary school completed	129	26	83	33	39	25	7	8
Tertiary completed	80	16	56	22	17	11	7	8
Retired	6	1	0	0	4	3	2	2
Employed/Self-employed	270	55	173	69	80	52	17	20
Pensioner	54	11	0	0	4	3	50	60
Unemployed	158	32	79	31	65	42	14	17
**Smoking history**								
Currently smoking	107	22	45	18	40	26	22	27
Not currently smoking	70	14	22	9	29	19	19	23
Never smoked	311	64	185	73	84	55	42	51
**Alcohol history**								
Never	367	75	189	75	109	71	69	83
Monthly or less	86	18	48	19	29	19	9	11
2–4 times a week	34	7	14	6	15	10	4	5
**Family history of hypertension or diabetes**								
Yes	188	39	95	38	69	45	24	29
No	300	61	157	62	84	55	59	71

### Lifestyle history and habits

Based on self-report, almost two-thirds (64%; 311/488) of participants had never smoked, 14% (70/488) were previous smokers and 22% (107/488) were currently smoking. Similarly, three-quarters (75%; 367/488) of participants indicated that they had never consumed alcohol, while 18% (86/488) had a drink once a month or less and 7% (34/488) had an alcohol-containing drink 2–4 times a week.

**FIGURE 2:**
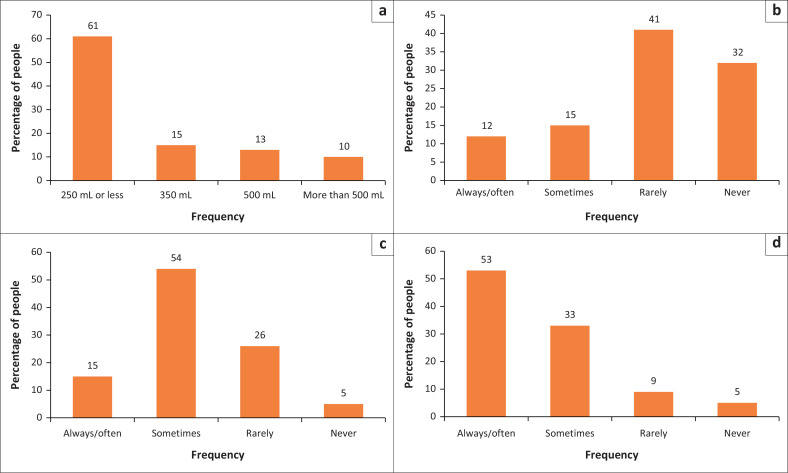
Prevalence of some unhealthy habits among participants enrolled in the study on the prevalence of multimorbidity in virally suppressed HIV-positive patients. (a) Sugary drinks consumption in a day. (b) Weekly usage of table salt or seasoning. (c) Weekly usage of processed food. (d) Weekly usage of salty sauce when cooking.

When questioned about weekly dietary habits, 12% (57/488) of participants used table salt, 15% (72/487) processed food and 53% (257/487) salty sauce when cooking always or often (Figure 2). In addition, 61% (269/441) of participants consumed sugary drinks of 250 mL or more daily. Weekly consumption of fruits and vegetables was daily for 34% (213/488) and 44% (213/488), and four or more times a week for 11% (53/487) and 16% (80/488) of participants, respectively.

**FIGURE 3:**
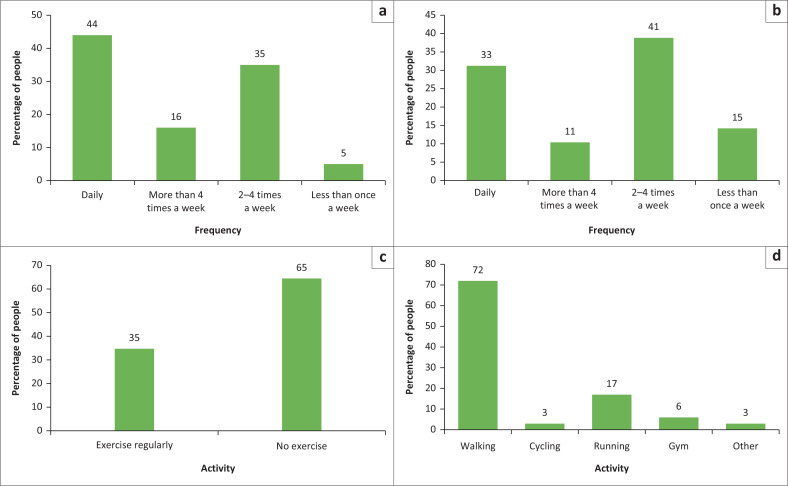
Prevalence of some healthy habits among participants enrolled in the study of the prevalence of multimorbidity in virally suppressed HIV-positive patients. (a) Weekly vegetable consumption. (b) Weekly fruit consumption. (c) Proportion of participants that exercise. (d) Types of exercise.

Self-report of regular exercise (more than twice per week) was 65% (319/488); walking and running (Figure 3) were the most common forms of exercise.

### Medical history and comorbidities

Approximately a third (38.5%; 188/488) of participants had a family history of hypertension, or diabetes or stroke (Table 1). For those for whom we had information on antiretroviral regimens, 41.0% were on first-line regimen. Participants were on their current regimen for a median of 6 years (IQR: 4–9).

Thirty-four percent (161/472) and 24.6% (116/472) of participants were overweight and obese. A total of 192 (39.3%) of participants had two (including HIV) or more chronic diseases. The most prevalent chronic disease among virologically suppressed PLWH was hypertension. Almost a third (32%; 158/488) of participants were diagnosed and receiving treatment for hypertension (Table 2). The second most prevalent was diabetes, with 5% (24/488) of participants being treated for diabetes. Four people indicated that they had depression requiring treatment, while five were on treatment for arthritis. Only one participant was receiving treatment for TB.

**TABLE 2:** Prevalence of comorbidities among virally suppressed HIV-positive patients in Limpopo.

Variable	Overall (*N* = 488)		40–49 years (*n* = 252)		50–59 years (*n* = 153)		60+ years (*n* = 83)	
	*n*	%	*n*	%	*n*	%	*n*	%
Hypertension	158	32.0	56	22.2	50	32.7	52	62.7
Diabetes mellitus	24	5.0	7	2.8	9	5.9	8	9.6
Chronic kidney disease	2	0.4	0	0.0	2	1.3	0	0.0
Stroke or cerebrovascular accident	3	0.6	2	0.8	1	0.7	0	0.0
Arthritis	5	1.0	0	0.0	4	2.6	1	1.2
Asthma (chronic obstructive pulmonary disease)	9	1.8	4	1.6	4	2.6	1	1.2
Cancer	2	0.4	0	0.0	1	0.7	1	1.2
Depression requiring treatment	4	0.8	1	0.4	2	1.3	1	1.2
Tuberculosis	1	0.2	0	0.0	1	0.7	0	0.0
Other	16	3.3	11	4.4	2	1.3	3	3.6

A multivariate analysis on the risk factors for hypertension showed that age 60 years and older (relative risk [RR]: 1.76; confidence interval [CI]: 1.29–2.30), overweight (RR: 1.32; CI: 1.03–1.69); obesity (RR: 1.69; CI: 1.32–2.17) and being diabetic (RR: 1.42; CI: 1.08–1.87) are associated with a higher risk of hypertension (Table 3). Gender, residential area (urban vs rural), level of education, employment status, smoking and alcohol history and other lifestyle habits were not associated with an increased risk of hypertension.

**TABLE 3:** Risk factors associated with hypertension among virally suppressed HIV-positive patients in Limpopo.

Variables	Univariate			Multivariate		
	RR	95% CI	*P*	RR	95% CI	*P*
**Age group (in years)**						
50–59 versus 40–49 years	1.2121	0.9568–1.5356	0.1110	1.1759	0.9343–1.4800	0.1673
60+ versus 40–49 years	1.9212	1.5584–2.3686	< 0.0001	1.7246*	1.2925–2.3013*	0.0002*
**Gender**						
Female versus male	1.0719	0.8705–1.3199	0.5132	-	-	-
**Body mass index**						
Underweight versus normal	0.7538	0.3877–1.4658	0.4049	0.7388	0.3987–1.3690	0.3362
Overweight versus normal	1.3565	1.0481–1.7557	0.0205	1.3187*	1.0283–1.6911*	0.0293*
Obese versus normal	1.7621	1.3774–2.2542	< 0.0001	1.6940*	1.3240–2.1673*	< 0.0001*
**Diagnosed with diabetes mellitus?**						
Yes versus no	1.9018	1.5468–2.3383	< 0.0001	1.4228*	1.0803–1.8737*	0.0121*
**Does the participant currently smoke tobacco/use any tobacco products?**						
No but ex-smoker versus never smoked	1.0746	0.8172–1.4130	0.6066	-	-	-
Yes versus never smoked	1.0140	0.7978–1.2889	0.9094	-	-	-

RR, relative risk; 95% CI, 95% confidence interval.

* , Statistically significant.

## Discussion

This study that assessed the prevalence of NCD and their risk factors in a cohort of HIV-positive adults aged ≥ 40 years, virologically suppressed in the Limpopo province demonstrated that there is a high prevalence of NCDs and their risk factors in this group of participants. Based on self-report, the identified risk factors included regular usage of table salt, intake of drinks high in sugar and processed food and smoking. Some participants had protective factors, such as never smoked, exercised, and consumed fruits and vegetables regularly. Risk factors that were associated with hypertension on multivariate analysis included age 60 years or older, overweight, obesity, and being diabetic.

The observed high prevalence of 1% – 32% of one or more chronic diseases in PLWH in this study is comparable to the findings from other studies that assessed the prevalence of multimorbidity among primary healthcare clinics attendees that found a prevalence of 3% – 87%, and that multimorbidity increased with increasing age.^12^ Some of the factors associated with multimorbidity were living in urban areas, lower level of education and obesity.^12^ Another study conducted in an urban area of South Africa found that 21% of PLWH also have a high blood pressure.^33^ The reported prevalence of the high blood pressure of 20% among PLWH is lower that the observed 32% in our study, and this could be as a result of including participants of younger age groups, while our study only included adults 40 years and older.^33^

The identified risk factors for NCDs based on self-report included smoking history, with 14% of participants indicating that they were previous smokers and 22% were currently smoking. The prevalence of smoking in this cohort is comparable to the findings from Dikgale Health Demographic and Surveillance System in Limpopo that found 13.7% of participants were current smokers.^34^ This is slightly lower that what was stated in the urban study where 27% of participants were currently smoking.^33^ Another study that assessed the prevalence of NCD risk factors in adolescents living with HIV, in Western Cape, South Africa, reported high levels of 31% and 29% of daily intake of refined sugar and sugar-sweetened beverages.^35^ The prevalence of obesity in this cohort of adolescents living with HIV was 37%, which is slightly higher than the 25% observed in our study of older adults.^35^ The management of obesity needs to be included in health services for both adolescents and adults.^36^ Integrating screening, testing, treatment, and care for chronic CDs and NCDs for PLWH that are older would be recommended.

The prevalence of protective factors such as daily consumption of fruits and vegetables was 33% and 44% in our study and 28% and 52% in the adolescent cohort.^35^ These data are similar to results from the Dikgale Health Demographic and Surveillance System in Limpopo where 88.6% of participants had low daily intake of fruits and vegetables.^34^ Therefore, in addition to integrated health services, there needs to be an emphasis on other disease prevention such as lifestyle changes that could prevent or reverse NCDs in the management of PLWH.

### Limitations

The limitations of this study include the retrospective review of routine paper-based medical records that resulted in 19% of participants not being included in the analysis due to missing data. Based on sample size calculations, the aim was to enrol 700 participants; however, this target was not reached due to resource constraints and insufficient data. Second, participants that are in regular contact with the health systems such as those with chronic diseases, might have received some educational information on healthy lifestyle habits which could have introduced social desirability bias. In addition, few risk and protective factors were included in the questionnaire, which provides a limited understanding of the prevalence of those factors. Data on people approached that declined to participate in the study were not collected. The results of this study cannot be generalised to all PLWH as only those 40 years and older, and only those with virologic suppression were included.

## Conclusion

This retrospective study that included a cross-sectional health questionnaire and minimal medical examination demonstrated that there is a high prevalence of NCDs and their risk factors among adults 40 years and older that are accessing ART in Limpopo. Integrated screening, testing, treatment, and care for chronic NCDs especially in older patients is recommended. There is a need for more research on the design and implementation of such programmes.
